# Introduction of DNA‐HPV test in the Brazilian cervical cancer screening program: When the idea is good but does not fit the budget

**DOI:** 10.1002/ijgo.70594

**Published:** 2025-10-14

**Authors:** Felipe Polido Urbano, Gabriel Russo Cury, Caio Rodrigo dos Santos, Daniel José Castilho da Silva, José Cândido Caldeira Xavier‐Júnior

**Affiliations:** ^1^ Pathology Department, Medical School Salesian Catholic University Center Auxilium (UNISALESIANO) Araçatuba São Paulo Brazil; ^2^ Pathology Department Institute of Araçatuba Araçatuba São Paulo Brazil; ^3^ Pathology Department São Paulo State University, UNESP Botucatu São Paulo Brazil

**Keywords:** cervical cancer, cervical smear, screening programs

## Abstract

**Objective:**

To evaluate the financial applicability of the DNA‐HPV test for cervical cancer screening in a developing country like Brazil.

**Methods:**

This was a retrospective, observational study based on documentary data, conducted to assess the cost‐effectiveness of cervical cancer screening strategies in Brazil. The study used national cervical cancer screening data collected between January 1 and December 31, 2020 for women between 25 and 64 years old, performing a cost‐effectiveness assessment of two different screening tests. No sensitivity analysis was conducted due to the descriptive nature of this preliminary cost assessment.

**Results:**

Based on the cervical smear, the Brazilian protocol has an estimated cost of US$117.5 million. In contrast, when using the HPV DNA test, the estimated cost is US$1.39 billion.

**Conclusion:**

The current use of the cervical smear is the least expensive and the only one that aligns with Brazil's financial resources allocated to health.

## 1 INTRODUCTION

According to data from the Global Cancer Statistics, in 2022 there were 348 189 deaths due to cervical cancer worldwide, corresponding to 3.6% of deaths among all types of cancer.^1^ In 2020, the WHO estimated 604 000 diagnoses of cervical cancer.^2^ In Brazil, data from the Instituto Nacional de Câncer (INCA) from 2023 indicated cervical cancer as the third most common malignant neoplasm among women, corresponding to 7% of all cancers, with an incidence rate of 17 010, despite the screening program offered by the Sistema Único de Saúde (SUS).^3^

Regarding estimates in Brazil, between the years 2000 and 2012, there was a growing trend of advanced cervical cancer diagnoses, resulting in 65 843 new cases.^4^ In addition, according to the 2022 INCA annual report, about 35% of cases are still diagnosed in advanced stages (stages III and IV).^5^

Until now, the Brazilian Cervical Cancer Screening Program is opportunist and based on conventional cytology. The target women are 25 to 64. Women can be submitted to cervical smear tests spontaneously without any invitation or restriction. The first two examinations are recommended annually. If both are negative, subsequent tests are indicated every 3 years.^6^ Screening stops at 64 years old if women have two negative tests in the previous 5 years and have no cancer history.

It is essential to highlight that the cervical smear sensitivity is around 51% while its specificity is around 98%, which is a good but imperfect examination.^7^ Possible explanations for the suboptimal performance of the current screening program include the low screening coverage rate, low‐quality control of preanalytical factors, low qualification of the professional who performs the examination, and frequency of representation of the transformation zone under expectations.^8^ Since 1999, Brazilian primary care has offered cytological and histopathologic examinations for cervical cancer prevention, guided by quality standards^9^ and despite the coverage target of 80%, in 2020, just 55% of the expected women were submitted to the cervical smear.^10^

According to data from the Sistema de Informação do Câncer (SISCAN), 3 754 408 new cervical smear examinations were performed between January 1 and December 31, 2020. Among those women, 741 215 (16.5%) were outside the target group,^5^ and only 62 493 women continued to follow the protocol, performing periodic examinations (every 3 years).^11^ It is estimated that less than one‐third of the tests were performed according to the official guidelines, and more than two‐thirds were unnecessary.^12^ Since the population projection carried out by the Instituto Brasileiro de Geografia e Estatística (IBGE) estimated for the year 2020 was around 61 742 997 women between 25 and 64 years old in Brazil,^13^ and the suggested screening coverage is 80%,^2^ we can infer low adherence to the current cervical cancer screening protocol.

Globally, cervical screening programs vary and have impacted different levels of early diagnosis and cancer prevention. Up to the last decade, most were focused on cytology. In the previous decade, cervical cancer screening has been modified by the incorporation of HPV deoxyribonucleic acid (DNA) testing, and the human papillomavirus (HPV) vaccination has become part of primary prevention with impacting cervical cytology incidence metrics.^14^

The guideline by the WHO recommends replacing cytology with the DNA‐HPV test for cervical cancer screening when adequate funds are available.^2^ It is crucial to assess how this shift can be implemented in countries like Brazil, where cytology‐based screening is the current option despite showing suboptimal performance.

## 2 MATERIALS AND METHODS

This retrospective observational study based on documentary data, with a descriptive quantitative approach, was conducted to assess the cost‐effectiveness of cervical cancer screening strategies in Brazil. The study used national cervical cancer screening data collected between January 1 and December 31, 2020 for women between 25 and 64 years old, performing a cost‐effectiveness assessment of two different screening tests.

For the costs in this article only the test kit prices in SUS in 2025 assuming screening coverage of 80% (WHO coverage recommendations) were considered; they did not include costs for personnel, laboratories, or any type of infrastructure. They were calculated in Reals (R$—the Brazilian real) and then converted to dollars to facilitate interpretation and comparability (currency on April 2, 2025, US$ 1 = R$ 5.66) based on SUS standard prices (Table 1), accessed in April 2025. No inflation adjustment or conversion to purchasing power parity was applied.

**TABLE 1:** Values of tests involved in screening.

Examinations	Price in US$	Suggested price
Cervical smear	$2.54^15^	‐
DNA‐HPV test	‐	$30.00^16^

The study was conducted using publicly available data from the Brazilian Ministry of Health, covering women aged 25–64 across all regions of Brazil. Data were extracted from official reports of the Brazilian National Cervical Cancer Screening Program and cost estimates were based on health system financial reports from DATASUS and similar public databases as SISCAN,^11^ Sistema de Gerenciamento da Tabela de Procedimentos, Medicamentos e OPM do SUS,^16^ IBGE,^13^ Departamento de Informação e Informática do Sistema Único de Saúde,^17^ and the INCA,^3^ accessed in April, 2025.

To simulate the financial impact of replacing the cervical smear with the DNA‐HPV test in the Brazilian scenario, population data between January 1 and December 31, 2020 for women between 25 and 64 years old was used. Although cervical cancer screening for cervical smear is triennial and quinquennial screening for DNA‐HPV test, in line with global screening recommendations, the hypothetical analysis in this study only considered the initial examination for women within the target population. The screening was applied to all women in the first year, regardless of previous examinations. There was no allocation of patients to groups, but rather comparative analytical scenarios between the two strategies. Two hypothetical screening scenarios were constructed: (1) screening the eligible population using conventional cervical smears, and (2) screening the same population using DNA‐HPV test. Finally, a cost estimate was provided for both screening modalities, based on one test per woman, without taking into account subsequent testing as recommended by each protocol. No subgroup analysis was conducted; cost projections were calculated based on national averages and not stratified by age, region, or socioeconomic status. Furthermore, there was not any type of clinical intervention, randomization, or direct interaction with patients, but it only involved a comparative economic evaluation of two screening strategies – cervical smear and DNA‐HPV test – applied to the same target population and no sensitivity analysis was conducted due to the descriptive nature of this preliminary cost assessment.

The analysis considering women aged 25 to 64 years, assumed universal access and compliance with the age range eligible for cervical cancer screening under the national guideline, and did not account for costs related to treatment, diagnostic follow‐up, or implementation. Population estimates for this age group were obtained from the 2020 Brazilian IBGE census.

Cost‐effectiveness was assessed by comparing the estimated national expenditure required to screen the target population using either cervical smear or DNA‐HPV testing, based on current unit cost estimates and screening coverage rates. No modeling of long‐term outcomes or QALYs was performed.

As the study used publicly available, de‐identified data, ethical approval was not required. A cost projection model was built using Microsoft Excel 365. The total cost for each strategy was calculated by multiplying the estimated number of women screened annually by the unit cost of each test. No discounting or sensitivity analysis was applied.

## 3 RESULTS

Assumptions to project the costs for cervical screening included the average cost per cervical smear test ($2.54) and per DNA‐HPV test ($30.00), a target population of 57 833 287 million women aged 25–64 years (IBGE projection database^13^) between January 1 and December 31, 2020 and assuming screening coverage of 80% (WHO coverage recommendations) a total of 46 266 630 examinations would be performed.

### 3.1 Costs of cervical smear screening

Within the Brazilian protocol, 46 266 630 cervical smears would be performed between January 1 and December 31, 2020 (considering 80% screening coverage of the female population aged 25 to 64 years) the total cost would be $117.5 million (Table 2).

**TABLE 2:** Table of screening methods. Screening value for each of the methods.

Screening methods	Total examinations	Screening cost
Cervical smears	46 266 630	$117 517 240
DNA‐HPV tests	46 266 630	$1 387 998 900

### 3.2 Costs of DNA‐HPV test screening

Within the Brazilian protocol, 46 266 630 DNA‐HPV tests would be performed between January 1 and December 31, 2020 (considering 80% screening coverage of the female population aged 25 to 64 years), reaching a total cost of $1.39 billion (Table 2).

The cost‐effectiveness of the present study was done through the total cost of the screening method/effectiveness, arriving at the number of efficient cases for screening, with effectiveness values of the cervical smear of 0.0175 (117 517 240 × 0.0175 = 2056551.7) and of the DNA‐HPV test of 0.0180 (1 387 998 900 × 0.0180 = 24983980.2), according to data described in Table 3.^18^ The same table shows how many positive cases are found for every 1 000 000 women screened, resulting in a difference of 500 positive cases between cervical smears and DNA‐HPV tests.

**TABLE 3:** Cost‐effectiveness between cervical smear and DNA‐HPV methods.

Diagnostic tests	Screening cost	Effectiveness^18^	Cost‐effectiveness	Number of cases/million
Cervical smear	$129 544 884	0.0175	2056551.7	17 500
DNA‐HPV	$1 387 998 900	0.0180	24983980.2	18 000

## 4 DISCUSSION

When comparing these screening methods in the Brazilian scenario for women aged 25 to 64, our results indicated that the current Brazilian protocol had the lowest cost. Its cost over the course of one screening per woman (considering 80% screening coverage of the female population) was US$117.5 million, significantly lower than the DNA‐HPV test (US$1.39 billion).

The cervical cancer screening based on the cervical smear, which is more affordable, has a referral rate of 2% and targets women aged 25 to 64 with a reduced interval between examinations. However, the SUS tracking protocol faces issues such as (i) lack of qualified professionals, (ii) inadequate slide quality control, (iii) increase in false‐negative results due to sample representativeness, (iv) lack of recall for new examinations and follow‐ups leading to low patient adherence, (v) an inefficient system that restricts access due to collection timings, (vi) insufficient coverage and (vii) no outreach to women who skip examinations.^19^ Therefore, given the current problems, it is evident that the protocol used for screening and early identification of cervical lesions has shown suboptimal results despite its significant impact during the national history.^20^ Aside from that, most of these problems will also influence any protocol performance, regardless of the type of examination chosen.

The Slide Quality Management Manual for Cytopathology Laboratories was created for cervical smear quality control.^21^ Despite the existence of this manual, an effective national control program with systematic slide reviews and periodic auditing has never been established from a national perspective.

Screening with HPV DNA testing by PCR identifies cases of cervical lesions caused by different subtypes of the HPV virus, but it is expensive and requires qualified technicians to perform. This challenge increase more because the SUS operates with decentralization as its principle, and each Brazilian state has its own unique tech and economic reality.

In 2020, in Brazil, the budget allocated to health was identified at US$ 150.46 billion, of which US$ 25.8 billion was allocated to primary care in the same year. When analyzing the budgetary impact through the percentage of primary care spending that each of the protocols, the cervical smear method would account for 0.455% and the DNA‐HPV test for 5.39% of the 2020 budget.

Previous data indicated that DNA‐HPV testing can be more effective.^22^ The cervical smear is less reliable in obtaining a negative result than DNA‐HPV test. However, it is more reliable in ensuring the absence of a high‐grade precancerous lesion. A series of critical clinical studies corroborated this finding conducted internationally.^23^

The cervical smear presents the best cost‐effectiveness of the cervical cancer screening protocols analyzed. It accounts for 2.05 million cases in terms of the protocol's value. On the other hand, the effectiveness values of the DNA‐HPV test (0.0180) were greater than those of the Brazilian (0.0175).

HPV infections are common and often self‐resolving, especially in young, sexually active women. Opportunistic HPV screening without a structured plan may result in unnecessary colposcopies, overtreatment, increased healthcare costs, and psychological stress, particularly in less educated populations. Therefore, a well‐defined cervical cancer screening program based on solid quality control protocols is crucial.

A recent guideline recommended DNA‐HPV tests for the SUS portfolio.^24^ It is unquestionable this is an achievement. Nevertheless, how it will work in this publication is still being determined. Considering all Brazilian disparities, including the cervical cancer screening scenario, it is challenging to find the logistic structure to support the examination for all target women. Otherwise, it does not describe who (public or private services) will run the tests and who will provide the material (kit) to collect cervical cells. Another important topic to consider is the availability of qualified human resources to run PCR and the available gynecologists in public services to meet the growing colposcopic demand.

In the vast majority of developing or undeveloped countries, such as Brazil, the cervical smear remains as the cervical cancer screening method. Even though the reduction of costs considering the treatment of advanced cases is a possible explanation to the introduction of the DNA‐HPV test, many other aspects must be evaluated and planned, such as the size of the target population of the country's screening protocol, how women will be approached and sought for screening, the availability of technological resources, materials and professionals (gynecologists to perform colposcopies and molecular staff to run the tests) at the national and not only regional level.^25^ Aside from that, other fundamental factors to ensure quality control processes ought to be explained. The portion of the public health budget calculated for the simple implementation of the DNA‐HPV tests did not consider other expensive and indispensable topics, such as the costs to implement quality control, personal training and equipment acquisition which could have a detrimental impact on other public health demands in Brazil. If the transition process is not well planned, we are at risk of increasing the costs of countries which do not have sufficient economic resources and reducing the real impact of DNA‐HPV test.

## 5 CONCLUSION

Although the DNA‐HPV test is superior for cervical cancer screening, its high cost could hinder its implementation in the current Brazilian context. New studies in distinct Brazilian regions are necessary to prove the DNA‐HPV test's performance in our regional realities. Independent of the test method, quality control programs, continuing education, the structure of the screening program, and the increment of the HPV‐vaccine campaign are the tools that can be used to set up cervical cancer screening performance in any country. Sadly, but perhaps for Brazil and many other developing or undeveloped countries, such implementation is a step that is not yet in line with their economic reality.

## AUTHOR CONTRIBUTIONS

**Felipe Polido Urbano:** Conceptualization, data curation, formal analysis, investigation, methodology, validation, visualization, writing and original draft preparation. **Gabriel Russo Cury:** Conceptualization, data curation, formal analysis, investigation, methodology, validation, visualization, writing and original draft preparation. **Caio Rodrigo dos Santos:** Conceptualization, data curation, formal analysis, investigation, methodology, validation, visualization, writing—original draft preparation. **Daniel José Castilho da Silva:** Conceptualization, data curation, formal analysis, investigation, methodology, validation, visualization, writing—original draft preparation. **José Cândido Caldeira Xavier‐Júnior:** Conceptualization, data curation, methodology, project administration, supervision, validation, writing—review and editing.

## FUNDING INFORMATION

None.

## CONFLICT OF INTEREST STATEMENT

None.

## Data Availability

Data sharing is not applicable to this article as no new data were created or analyzed in this study.
